# Sociocognitive and Argumentation Perspectives on Psychometric Modeling in Educational Assessment

**DOI:** 10.1007/s11336-024-09966-5

**Published:** 2024-04-03

**Authors:** Robert J. Mislevy

**Affiliations:** University of Maryland, Annapolis, MD 21409 USA

**Keywords:** assessment argument, automated scoring, measurement models, interactive tasks, sociocognitive perspective

## Abstract

Rapid advances in psychology and technology open opportunities and present challenges beyond familiar forms of educational assessment and measurement. Viewing assessment through the perspectives of complex adaptive sociocognitive systems and argumentation helps us extend the concepts and methods of educational measurement to new forms of assessment, such as those involving interaction in simulation environments and automated evaluation of performances. I summarize key ideas for doing so and point to the roles of measurement models and their relation to sociocognitive systems and assessment arguments. A game-based learning assessment *SimCityEDU: Pollution Challenge!* is used to illustrate ideas.

## Introduction

Significant developments are taking place in educational assessment and educational measurement: in technology, for assessments that can be interactive, immersive, simulation-based, created, and personalized, on the fly; analytic methods, in modeling and computation, learning analytics, machine learning, and natural language processing; systems and strategies to better integrate assessment with learning; and interest in higher-order capabilities, such as systems thinking and collaboration. I focus here on two foundational advances that help us put these developments to work effectively and validly, as they connect with longstanding concepts and principles from psychometrics. The advances are these:

- A sociocognitive psychological perspective, which concerns how people develop capabilities and use them to interact in the social and physical world.
- Assessment argument structuring, which explicates issues in design and inference in ways that a measurement paradigm alone does not.

I draw on three projects I have recently been involved with: *Sociocognitive foundations of educational measurement* (Mislevy, 2018) expands further on these two themes. The chapters of the edited volume *Computational psychometrics: New methodologies for a new generation of digital learning and assessment* (Von Davier et al., 2021) go more deeply into new analytic methods for measurement modeling and data analytics from this perspective. The *Handbook of automated scoring: Theory into practice* (Yan et al., 2020) provides further theory and examples on the evaluation of complex performances, connecting the concepts and methods of educational measurement with concepts and methods from data analytics and language processing to evaluate complex performances.

### Connecting Psychological Processes and Assessment Arguments

Samuel Messick (1994) proposed a way to begin thinking about assessment design:

*A construct-centered approach would begin by asking what complex of knowledge, skills, or other attribute should be assessed, presumably because they are tied to explicit or implicit objectives of instruction or are otherwise valued by society. Next, what behaviors or performances should reveal those constructs, and what tasks or situations should elicit those behaviors? Thus, the nature of the construct guides the selection or construction of relevant tasks as well as the rational development of construct-based scoring criteria and rubrics. *(p. 16)

Some notion of the nature of capabilities and how people develop them and use them is implicit in this quote. This is where a psychological foundation comes in. Note also that a construct construed in this way is historically and socioculturally located. It concerns regularities in behavior among people and situations, in some milieu of activity, as relevant to the purpose of the assessment. In any application, assessment designers need to determine how Messick’s general elements play out in the context at issue. Note finally that the quote is the core of an assessment argument, supporting both assessment design and score interpretation. We will see that there is more to it.

### An Example: SimCityEDU: Pollution Challenge!

While the conception I describe also applies to familiar forms of educational assessment such as multiple-choice items and written responses, I mean to highlight new forms, often digital. The following projects illustrate aspects of the ideas:

- Unobtrusive modeling of students’ proficiencies (aka “stealth assessment”) in computer game-based assessment with theory-based task design and a Bayes net student model updated by automated evaluations of students’ solutions (Ke and Shute, 2015).
- A scenario-based science assessment in which students work through menu-based branching conversations to predict the likelihood of a thunderstorm (Liu et al., 2016).
- A simulated science lab with theory-based investigations, affordances, and automated scoring procedures that generalize across tasks (Gobert et al., 2012).
- A tutoring system that automates evaluation, adapts problems, and provides interactive feedback for Newtonian physics, with an underlying measurement model to manage evidence and inference (Conati et al., 2002).

**Fig. 1:**
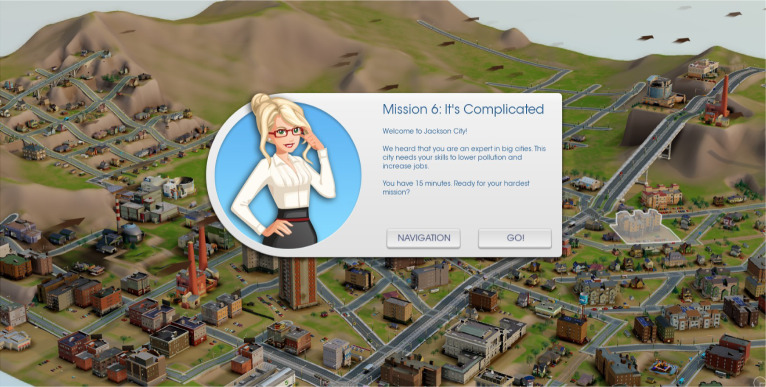
Initial view of Jackson Citycaption. *Source*: From Mislevy et al. (2014). Used with permission from the Institute of Play.

**Fig. 2:**
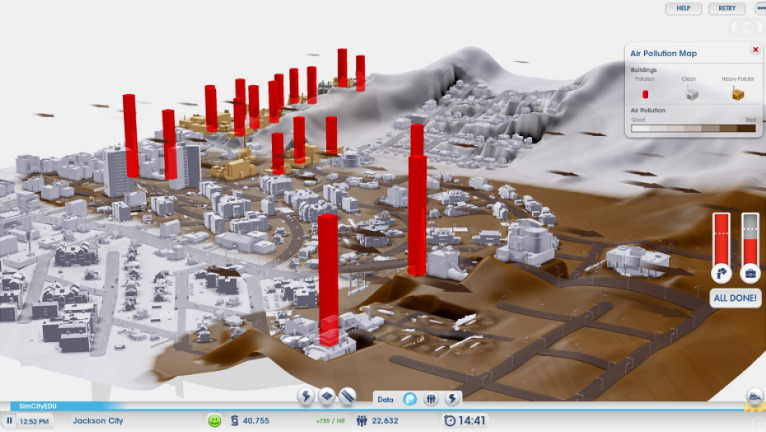
Use of a tool to monitor pollution production. *Source*: From Mislevy et al. (2014). Used with permission from the Institute of Play.

In this article, I will refer to a game-based assessment called *SimCityEDU: Pollution Challenge!* (Mislevy et al., 2014). *SimCityEDU* is a formative assessment, focused on systems thinking, embodied in a series of challenges in an environment based on the SimCity commercial game. It is designed around the more general five-level learning progression for systems thinking shown in Table 1, adapted from Brown (2005) and Cheng et al. (2010). Figures 1 and 2 are screenshots from one of its challenges, Jackson City, designed to provide an experience with an energy/pollution system that requires level 4 thinking to solve a problem and to educe evidence about a student’s thinking through their actions.

It is already apparent that its design reflects the Messick quote. The levels of the learning progression jointly bring in (1) the capabilities of a person in terms of thinking about a system, (2) key features of a system that underlie a situation involving that system, and (3) actions a person can take in interacting with the system. These relationships drive the technical aspects of evidence identification and measurement modeling.

**Table 1:** The systems-thinking learning progression.

Level	Competency level Description
1	*Students have a* *fragmented understanding of aspects of systems.* They may have partial knowledge of some of the definitions of system terms but cannot use them in a consistent or strongly coherent manner. While they can identify outcome variables (e.g., stocks that are explicitly part of the goal state), they are not able to track a causal link and they largely focus on macro-level directly observable variables. Their predictions and explanations are acausal, i.e., more assertions than cause-and-effect relations (e.g. “things happen because that’s the way they are.” Brown, 2005, p. 7)
2	*Students have an* *elemental understanding* (Brown, 2005, p. 7) of some aspects of systems—they can use models to represent simple, single cause-and-effect relations but without strong justification, i.e., they are still prone to common misconceptions, e.g., they tend to only relate macro-level, directly observable causes and effects rather than identifying hidden variables and factors. This is due in part to not being able to understand and analyze a system at different levels (Cheng et al., 2010). They are better at explaining than predicting
3	*Students have a* *locally coherent understanding of many aspects of systems.* Students can use system thinking terms to describe components and system relations in some contexts and use different representations. They can use models to represent bivariate cause-and-effect relations along with strong justifications. They can relate binary combinations of hidden and directly observable combinations, and even single causes to multiple effects. They are less prone to common misconceptions but still are limited to linear thinking with single causes (which may or may not be chained together.) They have a rudimentary understanding of negative feedback and can use it to explain and predict changes in the behavior of a system over time. They still are not able to consistently understand and analyze a system at different levels (Cheng et al., 2010)
4	*Students can relate* *multiple causes to multiple effects* as long as they behave in simple ruleful ways (e.g. cases in which all causes are needed for the effect to occur, cases in which all causes contribute independently to the amount of the effect as in Jackson City, etc.; that is, the causes are not emergent but are explainable in terms of the causal component parts.) This level is consistent with Brown’s (2005) conceptual depth level 4. Students can apply this scope of understanding within a wider range of contexts than in prior levels
5	*Students have a* *globally* *coherent understanding of many aspects of systems thinking in many contexts*. They can analyze of moderately complex system that includes multiple variables that may include hidden variables, feedback spread out in space and time, and emergent behaviors that require understanding a system at multiple levels, with multiple causes interacting to create complex emergent effects (corresponding to level 5 in Brown, 2005)

*Source*: From Mislevy et al. (2014). Used with permission from the Institute of Play.

**Fig. 3:**
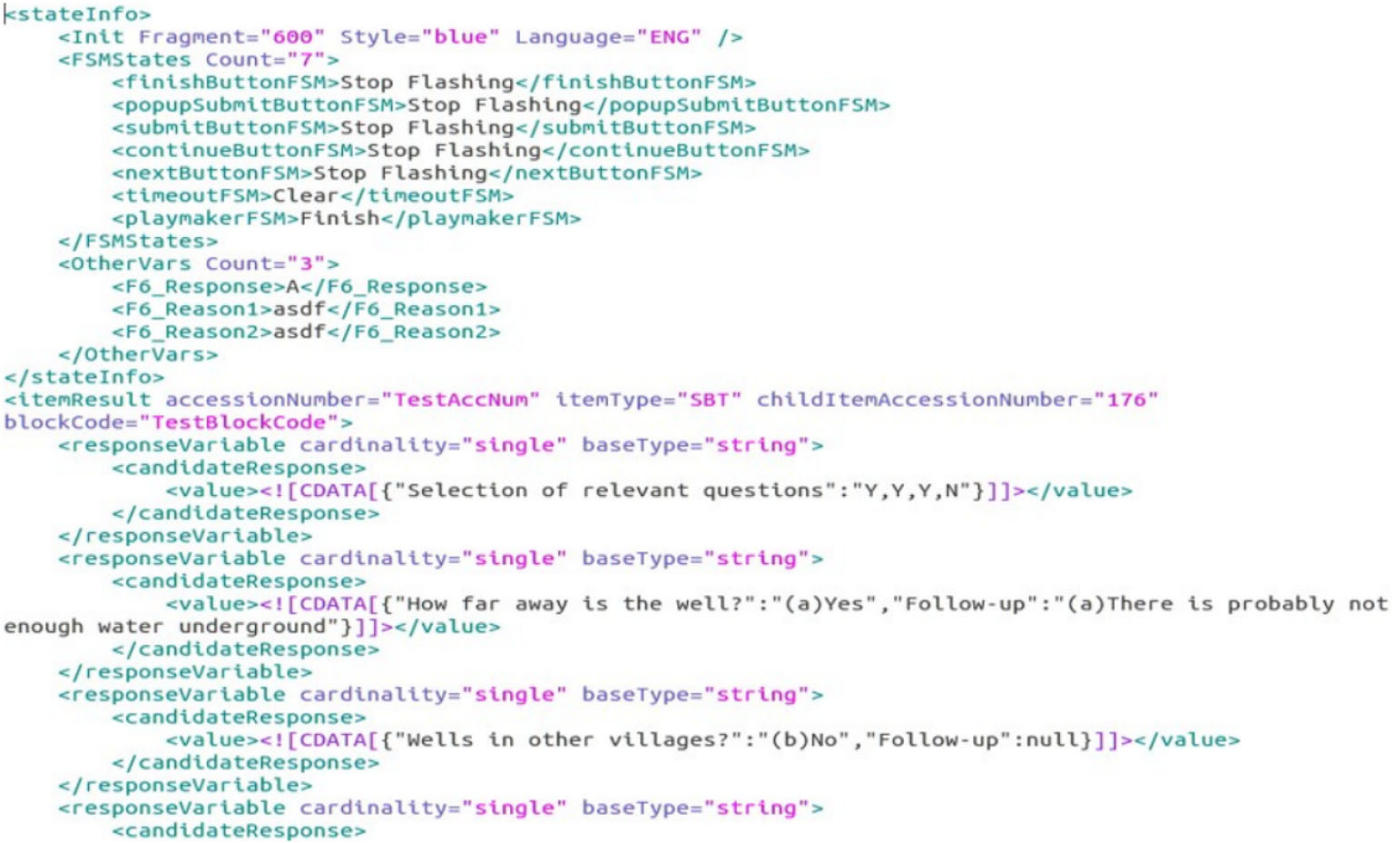
Log file data from Jackson City activity. *Source*: From Mislevy et al. (2014). Used with permission from the Institute of Play.

*SimCityEDU* does not consist of prepackaged items with easily identifiable and scorable responses. For example, a student tackling the Jackson City challenge to reduce pollution while maintaining electric power and commerce uses tools to explore the city, come to understand the problem, interact with the city through zoning and building actions, and examine their effects. Figure 3 shows a couple of seconds worth of data, from one student in one challenge. What does one do with data like this? How does one make sense of the evidence when different students can follow different paths and use different strategies?

## A Complex Adaptive Sociocognitive Systems Perspective

**Fig. 4:**
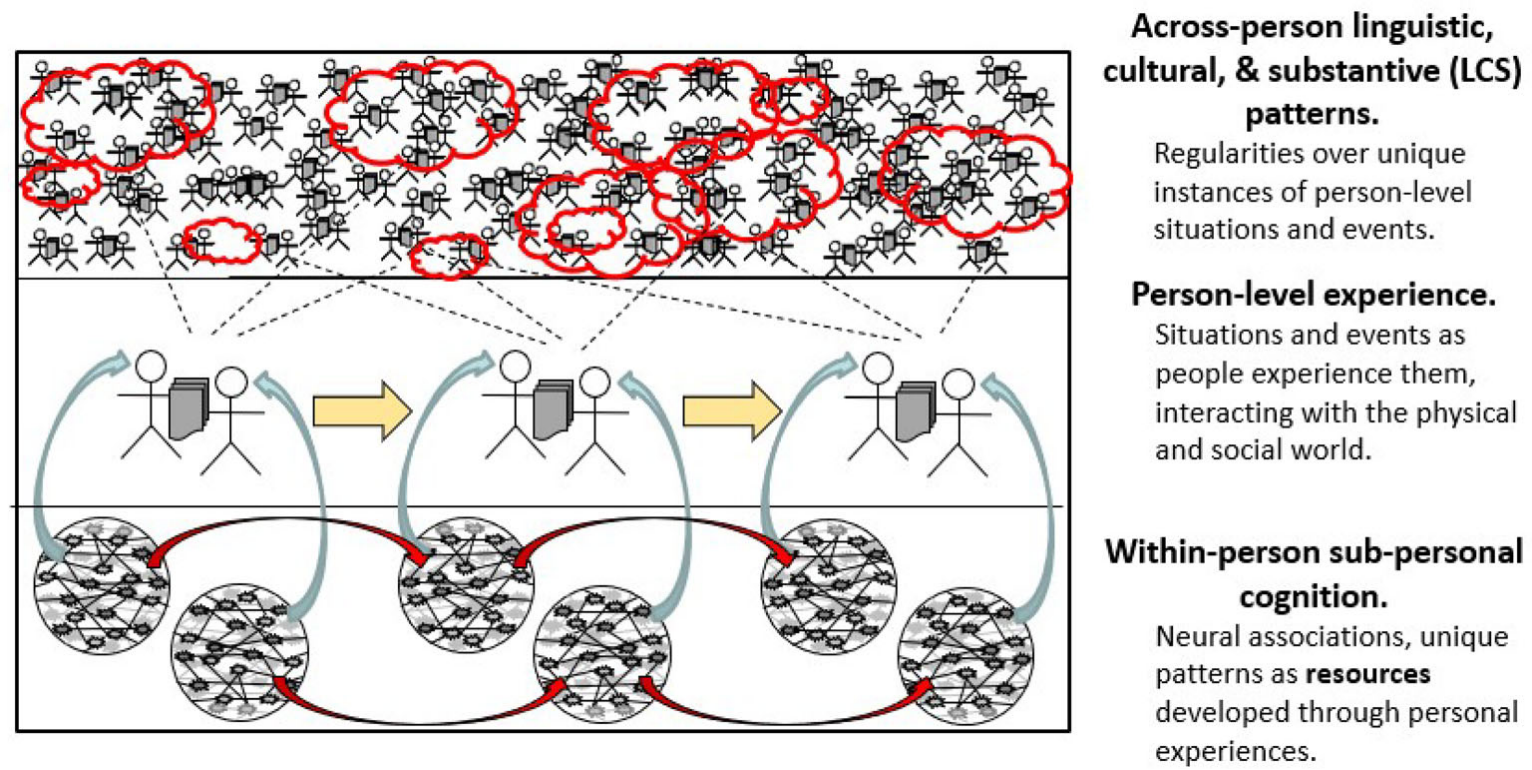
A complex adaptive sociocognitive system. *Source*: Adapted from Mislevy (2012). Used with the permission of Educational Testing Service.

Dennett (1969) uses the term “person-level experience” for situations and events as people experience them and think about them, as they interact with the physical and social worlds; having a conversation, giving a presentation, or taking a test, as examples. These activities unfold over time. They are depicted in the middle layer of Fig. 4.

For these interactions to be meaningful, much more must be going on at two levels, across people and within people. First, an interaction only makes sense by building on regularities across many such interactions, each unique, now and in the past–regularities that have to do with language, culture, and substance of an interaction, or LCS patterns for short. They are depicted in the top layer of Fig. 4. Every situation builds around many LCS patterns, of different kinds, at different grain sizes. Some of them a person is consciously aware of and thinks and acts through. Many more of them people are not aware of but think and act through them nevertheless. These regularities are culturally and historically contingent, meaning that LCS patterns can and do arise, evolve, and fade, and they vary over time and place, and across cultures and kinds of activities.

The existence of such regularities across people still isn’t enough. Individuals must be able to recognize LCS patterns implicit in a situation, blend them with the particulars of that situation, and have some options for what to do next. They must have developed relevant *cognitive resources* through their personal history of experience—traces and generalizations from those specific situations, which were built around their own particular mixes of LCS patterns (Hammer et al., 2005; Young, 2009). These resources take the form of patterns of associations in the neural network of an individual’s brain. They are depicted in the bottom layer of Fig. 4. While they are unique to a person, there can be similarities across peoples’ experiences with respect to LCS patterns, and therefore in the cognitive resources they develop through their own experiences, and enable them to interact meaningfully.

There are three things to note about Fig. 4: (1) This is a complex adaptive system (Holland, 2006), so concepts from that field prove useful to understand interacting social phenomena (Byrne, Byrne 2002). (2) LCS patterns and individuals’ resources are related through person-level activities and institutions, but they’re different kinds of things. LCS patterns are emergent regularities in ways of acting and thinking over individuals, across myriad activities and intersecting communities (Sperber, 1996), while cognitive resources are individuals’ attunements to such patterns as they have encountered instances of them through their experiences. (3) There are no constructs or measurement model $\theta$s, which are proficiency variables that are a hallmark of measurement models such as item response theory (IRT) and cognitively diagnostic models (Borsboom, 2008; Van der Linden, 2017).

Here are some key implications of this complex adaptive sociocognitive system for constructing assessments, assessment arguments, and psychometric and data analytic methods:

- Every person-level situation builds around LCS patterns of many kinds and levels, and this includes assessment situations.
- An individual’s experience of a situation assembles cognitive resources of many kinds, blended with features of that situation, much of which is below the level of consciousness (Kintsch, 1998).
- The cognitive resources each person develops are unique. They depend on personal history, in a person’s milieu of experience.
- Regularities *across* persons can arise due to similarities that shape the situations they have experienced. Thus arise patterns in people’s resources and actions. There are regularities and variation within and across people, and within and across situations.
- Such regularities and variation in a set of situations (e.g., tasks) as may arise—and as educators may arrange to arise—are the grist of constructs and of measurement modeling (see Gong et al., 2023, for an agent-based modeling illustration of the relation between sociocognitive processes and the parameters of an item response theory model).

## Assessment Arguments

This section outlines the form of an assessment argument, discusses how it is fleshed out from a sociocognitive perspective, and extends the argument structure to interactive tasks.

### The Basic Structure of Assessment Arguments

**Fig. 5:**
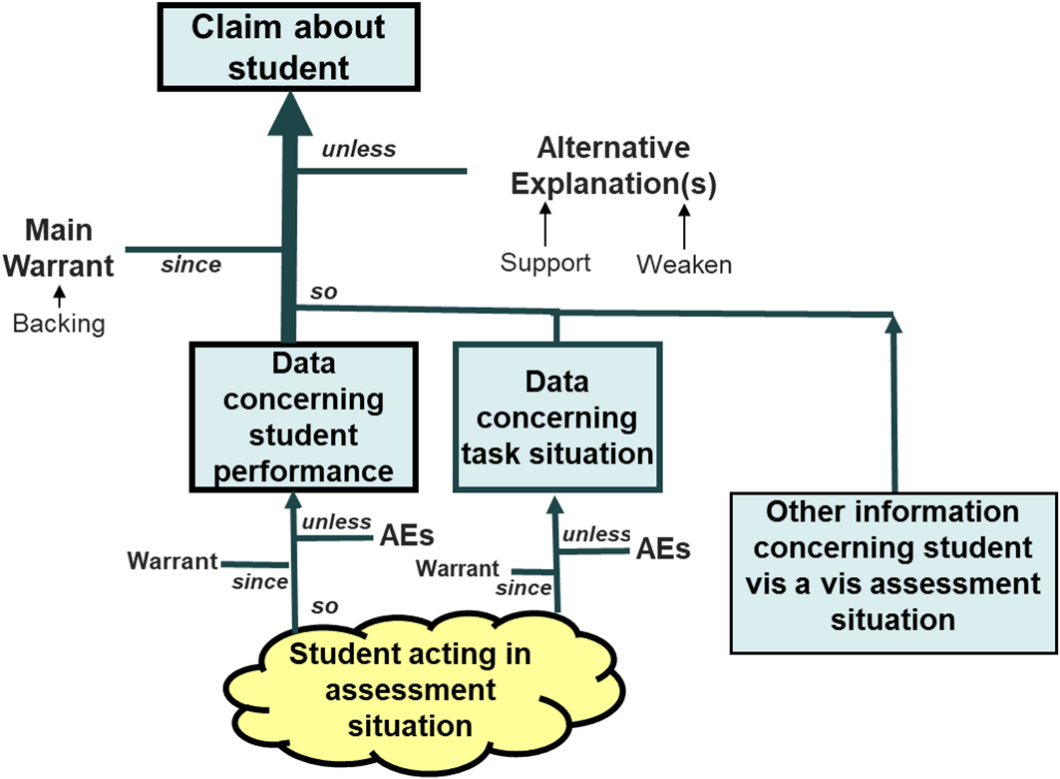
The basic structure of an assessment argument. *Source*: From Mislevy (2012). Used with the permission of the Board of Regents of California.

Figure 5 depicts the basic structure for an assessment argument. It captures the relationships expressed in Messick’s quote, using concepts and representations developed by Wigmore (1913) and Toulmin (1958), and modernized by contemporary evidence scholars such as Anderson et al. (2005) and Schum (2001)*.*Cronbach (1988), Messick (1989), Bachman and Palmer (2010), Kane (1992), Shepard (1993), and others have gainfully viewed assessment in terms of argument as concerning validity, and Mislevy et al. (2003), National Research Council (2001), and Wiley (1991) and others have done so concerning assessment design. Further extensions have addressed incorporation with learning models (Arieli Attali et al., 2019), assessment embedded in digital games (Ke and Shute, 2015), and affordances provided from digital environments (e.g., Behrens et al. 2012).

In an assessment of any type, a user (perhaps a teacher, a student, or an admissions officer) desires to make a claim about a person (perhaps a student or themself) in terms of some construal of capabilities of interest—constructs—based on some data. The same basic structure applies to assessments cast in any psychological perspective, including those under which educational measurement originated, namely trait, behavioral, and more recently, information processing. Greeno et al. (1997) argued that the sociocognitive perspective encompasses these other perspectives as special cases. I have discussed how, consequently, assessment arguments based on a sociocognitive perspective can similarly encompass arguments based on the other perspectives (Mislevy, 2018, Chapter 3–5).

When an analyst uses measurement models, the claim is represented in beliefs about the values of proficiency variables $\theta$. (Yes, I did say there are no $\theta$s in the actual complex system; I will return to this point presently.) The warrant is the set of beliefs and hypotheses that support this reasoning, such as generalizations, experience, scientific theories, and, in particular, psychometric measurement models. A central component of the main warrant in *SimCityEDU* is that a student able to reason at a given level in the progression in this context can generally reason at that level to tackle a particular challenge in which the underlying system and problem require it.

Alternative explanations are ways that despite the backing, a claim might not hold in a given case, even when backing supports the warrant as a generalization. As what Toulmin calls an informal argument rather than a logical argument, exception conditions can exist in an assessment argument. For example, in *SimCityEDU*, a student who does reason at Level 4 in some contexts might not do so in the Jackson City task because he is unfamiliar with the interface, misses some necessary information, or misunderstands the problem context.

Three kinds of data go into the main assessment argument, as shown in the three lower boxes. The first is aspects of a person’s performance. This is not directly observed, but rather it is an evaluation of a unique performance, the cloud representing a person saying, doing, or making things in an assessment situation. The arrow from the cloud to the performance data indicates a sub-argument for the inference from the observation to this data, through the construal of the construct. Alternative explanations may threaten that reasoning. I will return to how this sub-argument can become more complicated for the broader range of environments and interactions that assessment situations can comprise.

The next kind of data is features of the situation, because interpreting actions only makes sense in light of a situation—in evidence identification, as construed through the construct (which may not be the same as a student’s construal; in some cases, the intended identification is an inference about the student’s construal). It too is a sub-argument, with a warrant as to why the task situation satisfies the requirements of the main warrant to provide the desired evidence, and alternative explanations to investigate. In multiple-choice items, these features are built in by the test developer. In an interactive assessment, some features are built in, like the underlying system in a *SimCityEDU* challenge, but other features can be tailored to a student, and still other features of a situation a student is working in will emerge as the interaction between the system and the individual unfolds.

The third kind of data is additional knowledge about an individual in relation to the situation, because this knowledge conditions the inferences one can draw and the alternative explanations one must consider. Of all the many LCS patterns that are involved in the task for perception, action, and performance, any of them that are necessary but ancillary to the targeted construct can hamper some students and thus generate alternative explanations. The same performance in the same task holds different evidentiary value for you, for example, for inference about a student in your classroom when you know what they’ve been studying than it does for a user who doesn’t have this knowledge.

### Assessment Arguments from a Sociocognitive Perspective

Assessment argumentation from a sociocognitive perspective has the same structure as under trait, behavioral, and information-processing perspectives that measurement modeling evolved under, but now every element is further informed by a sociocognitive perspective (Mislevy, 2018, Ch. 4 & 5). Assessment claims can still be organized around such constructs, but analysts and users are aware that constructs are elements of the model the analysts and users are employing, not an existing well-defined attribute possessed by a student. That is, they are external actors’ characterizations of students in terms of behavioral consistencies as the designers and users construe them. This construal, as well as evidence about such claims, is embedded in the designer’s sociocultural milieu, which need not correspond well to that of various students, in potential ways that bring about alternative explanations. Different actors who work from different psychological perspectives, have different purposes, or operate in different milieus may reason through different constructs.

Warrants framed in terms of constructs are to be understood in terms of resources and LCS patterns. When an argument is cast in terms of trait, behavioral, or information-processing perspectives, the warrant includes the presumption that such an approximation suits the contexts, populations, and purposes at issue. Backing includes theory, research, and experience that ground this component of the warrant for the application at issue.

Note that while such backing may support the warrant as generally applicable, it may not hold for given individuals, groups, or populations in the application at hand. Analysts and users become aware of the importance in the argument of the dependence of contexts and the interplay with students’ histories of learning and current performances. The analysts and users are thus alerted to alternative explanations that arise from atypical student backgrounds or LCS patterns necessary but ancillary task demands (Messick’s sources of construct irrelevant variance). For example, in measurement modeling the presence of differential item functioning (DIF) and person misfit suggest that an alternative explanation may be at play to cast doubt on the usual interpretation of scores.

Evaluation of *performances* seeks evidence of students’ attunement to features of targeted LCS patterns and the activities they draw on, as suggested in their actions. The warrant in this sub-argument says why the evaluation procedure generally provides data for the claim about the construct; alternative explanations range from incorrect answer keys in multiple-choice items, to aberrant human ratings, to missing unusual but insightful problem-solving sequences in a problem-solving situation.

An interpretation of a performance depends on an evaluation (perhaps implicit) of *features of the task situation*, because moment-to-moment actions in situations are evaluated in light of targeted practices and LCS patterns. This can require a much finer grain size for interactive and collaborative assessments than familiar ones. This is a grain size that cognitive modeling and situative psychology address naturally and are employed explicitly or implicitly in automated evaluation procedures (Yan et al., 2020).

**Fig. 6:**
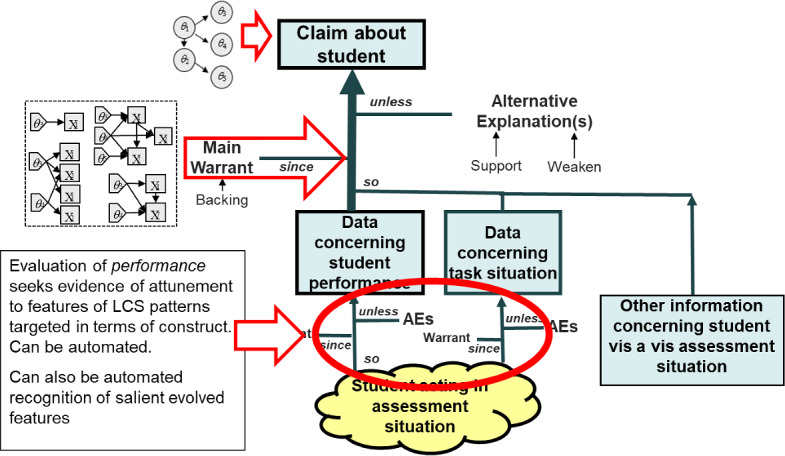
Locations of psychometric models and evidence-identification methods. *Source*: Adapted from Mislevy (2012). Used with the permission of the Board of Regents of California.

Figure 6 looks ahead to the principal places where psychometric measurement models fit into an argument when instantiated as an operational assessment. When claims are framed in terms of constructs and approximated in terms of values of $\theta$s in a measurement model, the conditional probability distributions from hypothesized $\theta$s to observable *X*s are an essential part of the reasoning, hence the warrant. A user reasons *as if* students possessed attributes $\theta$ and those $\theta$s caused performance (Mislevy, 2018a). Evaluations *X* of salient data features (indicated by the oval in the figure) are obtained by evaluation procedures such as human ratings, data mining, machine learning, feature detectors, natural language processing (NLP), etc. I will return to this topic presently. Reasoning then flows back up through the measurement model conditional distributions $p\left( x \vert \theta \right)$ via Bayes theorem to update belief about $\theta$. The warrant is that this measurement model approximation is adequate for the purpose at hand. Corresponding alternative explanations that arise are that the model does not fit sufficiently well to do so for an individual, for certain groups, or perhaps for anyone.

**Fig. 7:**
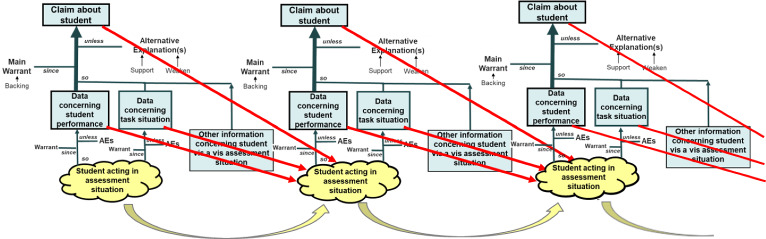
A sequence of dependent arguments as applied to an evolving performance. *Source*: Adapted from Mislevy (2018a). Used with the permission of Educational Testing Service.

### Extending the Structure to Interactive Tasks

The basic structure discussed above suits a static task with a simple response, but not one with interaction and change during the performance, like a *SimCityEDU* challenge. This state of affairs is suggested by a continuous series of dependent situations and dependent argument structure, as depicted in Fig. 7.

The situation in each new moment depends on the preceding situation and a person’s previous actions, and perhaps the preceding state of knowledge about $\theta$ (as in adaptive testing). New information can be obtained about both the new situation and the new actions. The snippet of data from *SimCityEDU* in Fig. 3 traces parts of this activity. Such data can be incorporated into the accumulating data stream for identifying patterns of actions across the evolving situation that bear on the student’s use of certain knowledge, tactics, or strategies, using “feature detectors” for example (Paquette et al., 2013). The evidence for construct-based claims is expressed in terms of estimates or posterior distributions for components of $\theta$. It may be posited that there is no change in certain $\theta$s during the course of observation while others change as a student learns through the experience. Different combinations of $\theta$s could be relevant in different situations that emerge.

## Measurement Models from a Sociocognitive Perspective

The previous section located measurement models and automated scoring in the assessment argument. The two following sections connect measurement models and automated scoring back to complex adaptive sociocognitive systems.

### Relating Measurement Models to Complex Adaptive Sociocognitive Systems

**Fig. 8:**
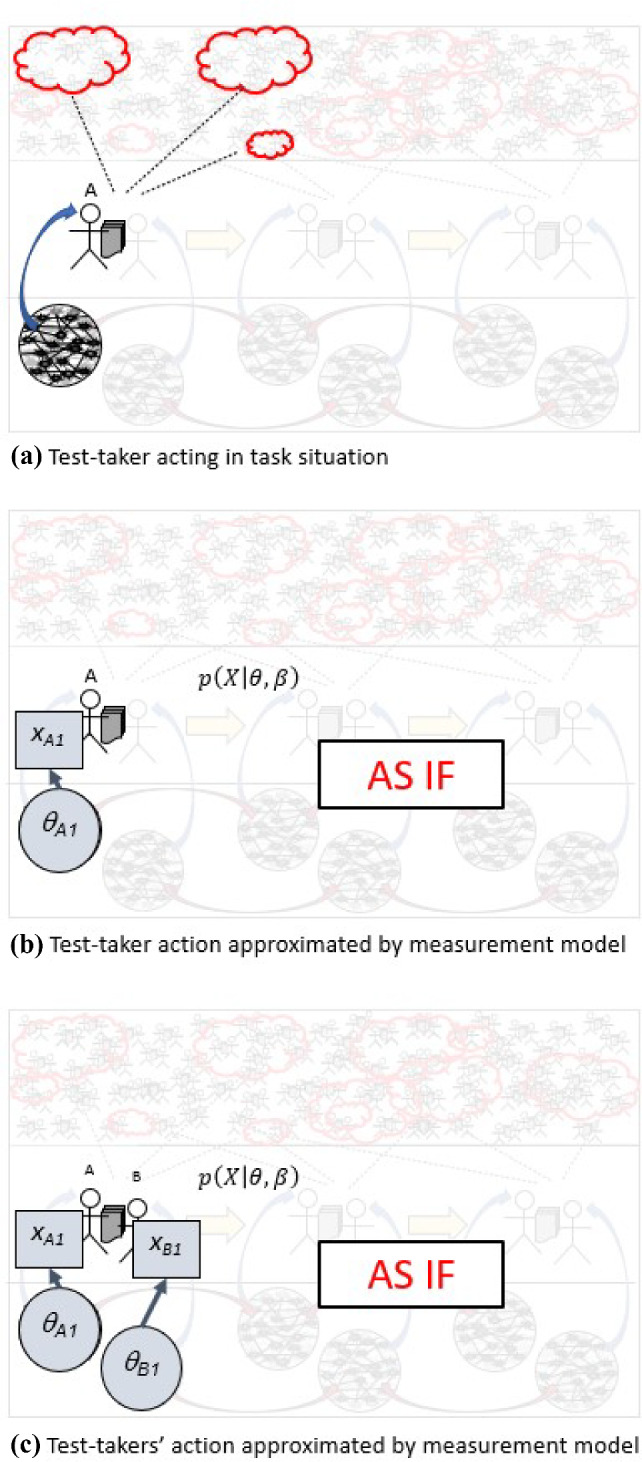
Approximating relations between capabilities and actions with a measurement model.

Panel a) in Fig. 8 suggests what happens when a test-taker interacts with an assessment task. The situation, as seen from the outside, builds around some LCS patterns, combined into an environment meant to evoke some activity of interest, in order to evidence some capabilities of interest, as construed through a construct, as expressed in values of person variables in measurement models. In contrast, what is happening in the world is that the test-taker tries to understand and act accordingly, through whatever unique, personal cognitive resources they bring to the situation—cognition and action quite distinct from the model, the $\theta$s, the probability structure.

Educational measurement modeling can be viewed as approximation relative to overall patterns within and across people and task situations, arising from a milieu of the actions of some collections of people and situations of interest (Fisher, 2017). Depending on the patterns, the populations, and purposes, apt models could include IRT, univariate or multivariate, or be structured around task or population features. They could be cognitive diagnosis or latent class models, and they could be expressed in the form of Bayesian inference networks. They could be mixtures of such models, and they could address different aspects of performance in varying combinations. The (Von Davier et al., 2021) *Computational Psychometrics* book discusses some more recent models.

I argued in *Sociocognitive Foundations of Educational Measurement* that from the perspective of model-based reasoning (Giere, 2004), between-persons educational measurement models are mathematical structures for expressing regularities and variabilities among persons, actions, and situations, shaped by theory and experience, built to serve understandings and purposes in contexts. From the perspective of probability-based inference, between-persons educational measurement models are Bayesian exchangeability structures (De Finetti, 1974) for managing evidence and inference in assessment. From a philosophical perspective, the $\theta$s take constructive-realist (Messick, 1989) interpretations as regularities associated with persons in the instantiated model over some populations and both assessment and non-assessment situations. It is not that constructs, and by extension $\theta$s, are real* per se,* but they do describe aspects of an ensemble model for patterns in actions, which are real, that arise from LCS patterns and practices in some milieu, which are also real, and within-person cognitive patterns, which too are also real, for functioning in that milieu. From a sociocognitive view, the sociohistorical locality and ongoing evolution of LCS patterns regarding education in culture will constrain the range and extent to which the forms and parameters of a model are suitable.

As a simple example to illustrate ideas, the Rasch IRT model for right/wrong test items addresses the observation that some people may tend to do better than others and some items are harder than others. The point here is just to relate measurement models in general to the argument structure and complex adaptive system diagram. Both the latent variable component $\theta$ and the data variable *X* of this model are quite simple: $\theta$ is a single continuous real-valued variable with higher values giving higher probabilities of a correct response, and *X* for any given item is a 0/1 response, however determined. This framing is shown in Panel b. Its parameters associated with people—$\theta$s—and parameters associated with items—$\beta$s—give a first approximation to the details of a matrix of 1’s and 0’s. Salient features *X* of the performance are identified and characterized in ways I discuss more generally in a following section. The measurement model gives us probability distributions of possible values of *X* conditional on possible values of $\theta$ and $\beta$; that is, $p\left( x \vert {\theta ,\beta }\right)$. The $\theta$s are the vehicle for model-based score interpretations and subsequent uses. A sociocognitive perspective cautions analysts that the overall patterns might differ with different collections of persons or situations. They are therefore on the lookout for systematic discrepancies for individuals or between groups that would suggest that alternative explanations hold for systematic patterns beyond those that a posited model can capture.

Panel c) adds another student taking the same assessment. Values of the same variable $\theta$ are used to characterize the capabilities of both students, and values of the same variable *X* are used to characterize the salient features of the responses of both students. Their cognitive resources may differ in a million ways and their performances may also differ in a million ways. But under the model, any differences in their performances are approximated as best as can be with only the possible values of *X*, and the differences in their capabilities as best as can be with only the possible values of $\theta$.

The analyst then reasons provisionally *as if* this model were true, and draws inferences in terms of $\theta$ based on values of *X*. This is why the model is explicitly part of the warrant, and why it opens the door to alternative explanations. Of course the model is wrong, but the issue is whether it’s good enough for the interpretations, the students, and the intended practical or scientific uses. The practical questions are then through which models, for what purposes, with which populations, facing what alternative explanations, is this approximation defensible? In a word, validation.

**Fig. 9:**
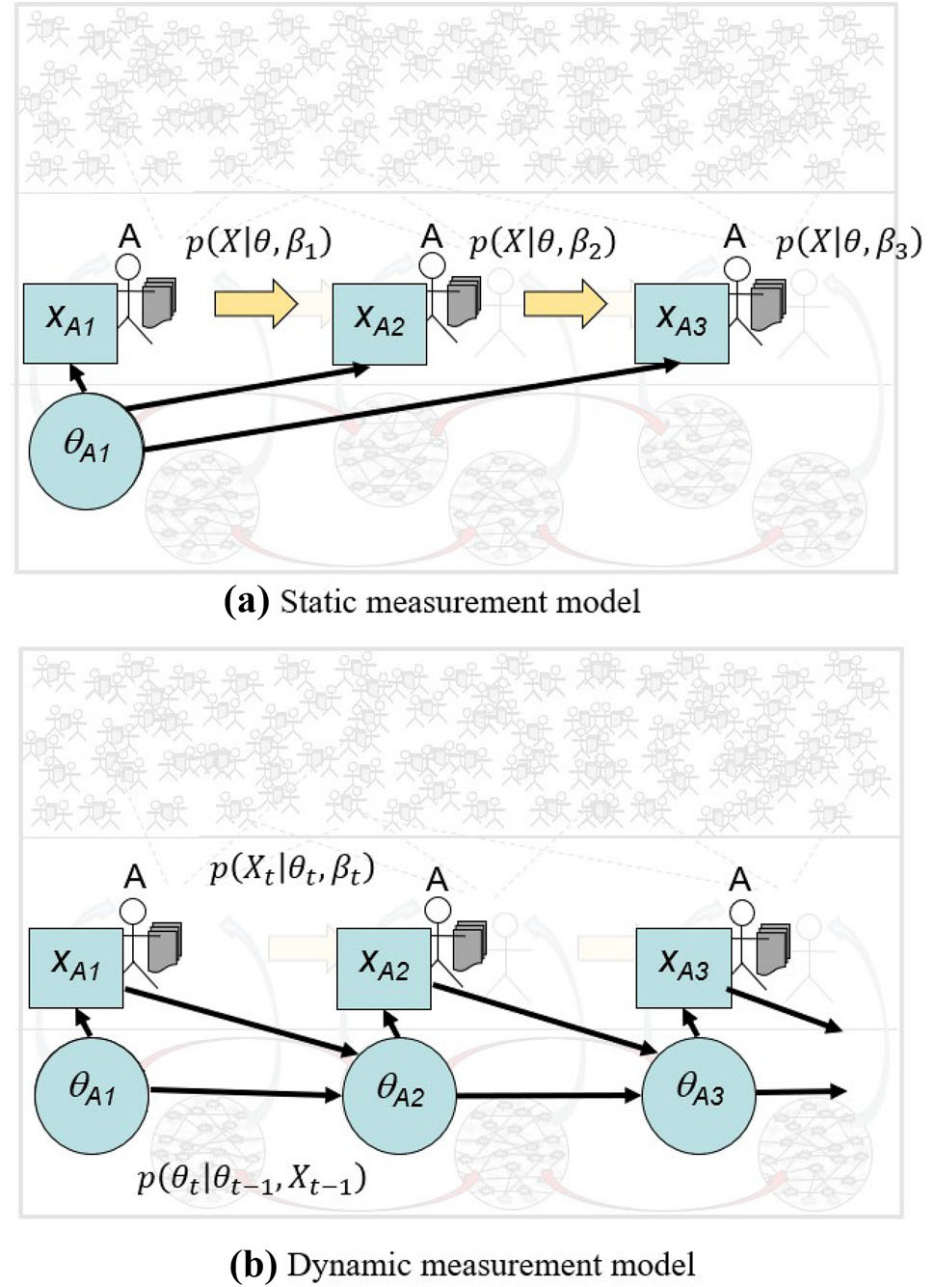
Static and dynamic psychometric models approximating assessment performance in a complex adaptive sociocognitive system.

By designing, contextualizing, tailoring to circumstances and populations, and recognizing then averting, mitigating, or detecting when alternative explanations hold, assessors can in fact sometimes create an assessment system in which users can more or less reason as if the constructs correspond directly to attributes of individuals and scores are measures of them. With familiar assessments, some of this pragmatic reasoning is built into good assessment practice, and some comes in by taking contexts, purposes, and populations into account in the task design, performance evaluation, and model construction. The more novel the assessment, the more useful an explicit framing like this becomes (Andrews-Todd et al., 2021), as with “stealth assessment” in game environments (Rahimi et al., 2023). It is equally useful when you are using an established assessment with a different interpretation, a new purpose, or a changing population (e.g., Fulcher & Davidson, 2009).

### Modeling Multiple Observations

In large-scale testing, the usual assumption has been that the changes in a person’s capabilities of interest are negligible while they interact with the assessment. Panel a) of Fig. 9 shows how the IRT measurement model framing plays out. The same but unknown value of $\theta$ for a person is presumed at all time points. The response spaces and situation features can vary across observations/tasks but are again characterizable only within the predetermined definitions of the respective *X* and $\beta$ variables.

If it is relevant to model $\theta$ as changing through the experience (which is the whole point in learning systems) one needs a model that takes this into account. Mathematical psychologists developed dynamic models in the 1950s; later came production-rule learning models, and currently, there are models such as dynamic IRT (Glas and Verhelst, 1993), Bayesian model tracing (Desmarais and Baker, 2012), and partially observed Markov processes (Halpin et al., 2021). Panel b) of Fig. 9 shows a structure in which a person’s $\theta$ at a given time point depends on both their previous $\theta$and their performance at the previous time point.

## Evidence Identification from a Sociocognitive Perspective

Now let’s look more closely at the rationale and the processes of identifying and characterizing evidence from a more complicated, interactive performance in a possibly-evolving situation. Ultimately an analyst wants to interpret actions in terms of evidence for claims about constructs, which can be instantiated in terms of perhaps multivariate proficiency variables $\theta$ in a latent variable model. This section describes in general terms a hierarchical evidence-identification structure as it applies to a given performance. The following section shows how it applies in a *SimCityEDU* challenge like Jackson City.

**Fig. 10:**
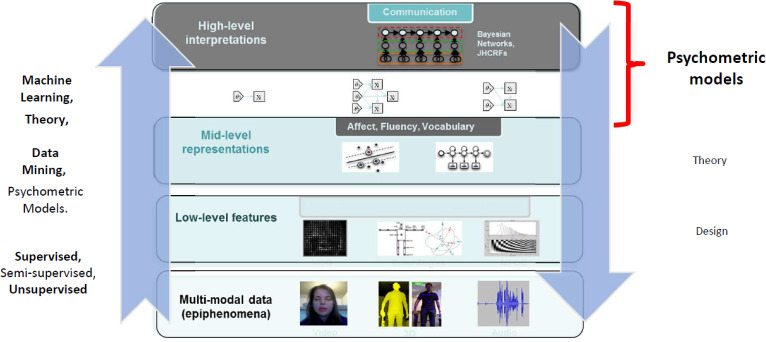
Hierarchical evidence and task-feature evaluation. *Source*: Adapted from Khan (2017). Used with the permission of Educational Testing Service.

What is initially captured in a complex performance as with a simulation- or game-based task is low-level data such as mouse clicks, drag-and-drop screen locations, and objects and connections in a construction. Modeling directly from the lowest-level observations to the highest-level constructs usually does not work well. For this reason, multiple layers of processing are typically employed in interactive assessments of any complexity. Figure 10 depicts a generic evidence-identification process, which allows for a sequence of successively refined processing stages: targeted latent variables at the top and lowest-level data at the bottom. This layering may be explicit, as with feature detectors at the lowest level providing input to a Bayesian inference network, or implicit, as with neural networks with multiple hidden layers (de Klerk et al., 2015; Yan et al., 2020).

Many evidence-identification techniques are being employed today in various assessment products and projects. At the left of the layers in Fig. 10 are some of the more bottom-up procedures that are used, including data mining and machine learning. The *Computational Psychometrics* book and the *Handbook of Automated Scoring* mentioned earlier provide many details and examples. At the right at some of the more top-down ones, like psychometric models and theory-based designed-in situation features and affordances to bring about evidence-bearing opportunities. Note that LCS patterns—features of situations, meanings, and actions—are involved in designing the interface, underlying system, and affordances. LCS patterns are involved also in recognizing patterns of action at a finer grain size to parse and evaluate evidence of test-takers’ capabilities.

**Fig. 11:**
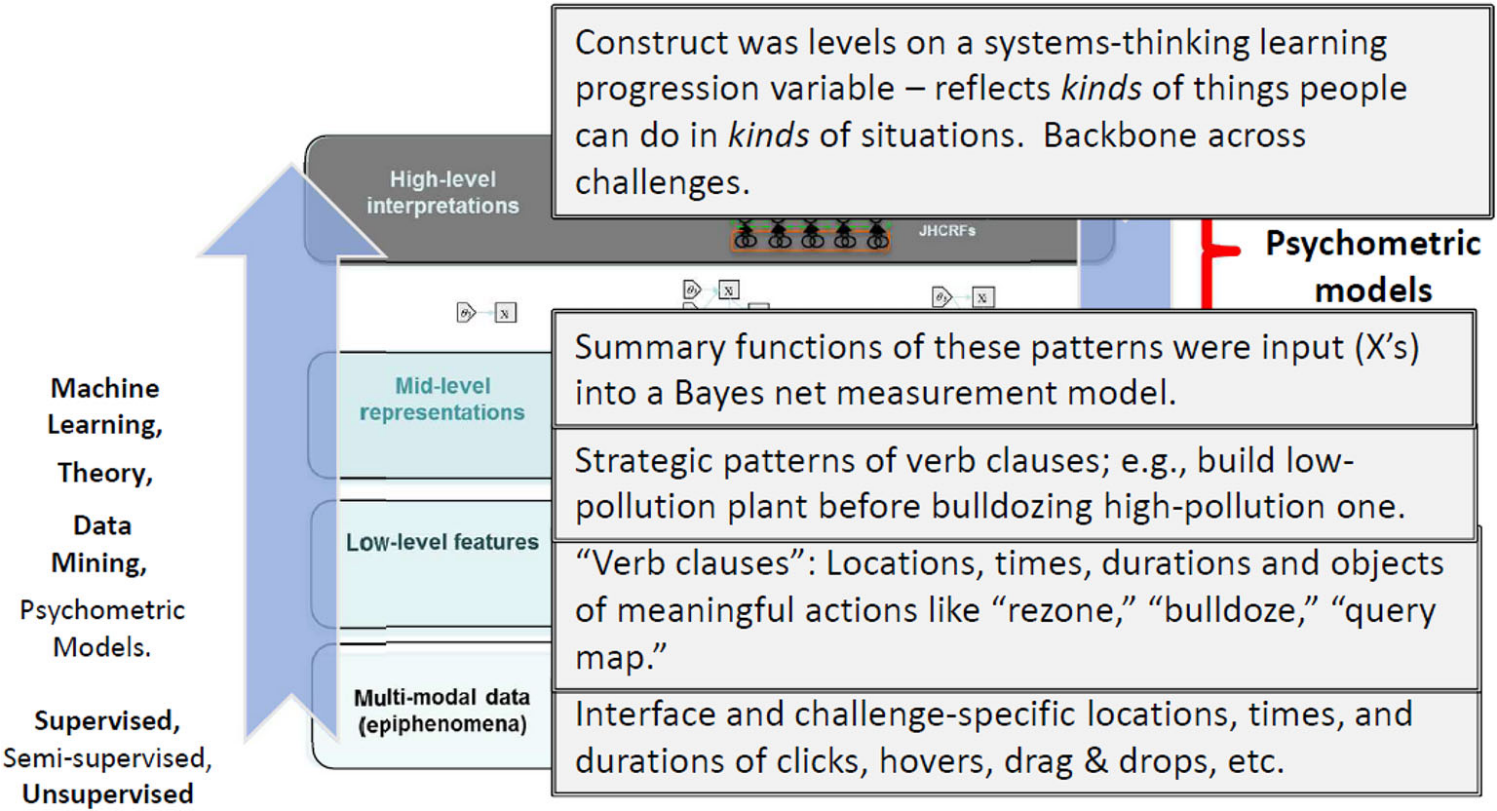
Evidence identification processes in SimCityEDU. *Source*: Adapted from Khan (2017). Used with the permission of Educational Testing Service.

In operation, low-level data is initially captured, and reasoning is successively upward. Moving up each layer is through reasoning that also can be analyzed in the same argument structure I have been using: What is the data coming in at that step, what is the intermediate claim coming out and being passed up to the next layer, what is the warrant for this stage and how well is it backed, and what alternative explanations is it vulnerable to? With inspectable models, one can examine the procedures and the reasoning using the structure of an assessment sub-argument with its substance as particularized LCS patterns.

## Evidence Identification and Measurement Modeling in SimCityEDU

### Evidence Identification

Figure 11 overlays the generic evidence-identification figure with the stages employed in the Jackson City challenge. The highest level is a summary of the salient aspects of the performance, as the *X* variables as evidence from this challenge about the targeted construct, operationalized as $\theta$ in an ordered latent class model defined by the systems-thinking learning progression (Table  1). That construct was levels on a systems-thinking learning progression variable, which reflects *kinds* of things people can do in *kinds* of situations. In a given performance on a given challenge, like Jackson City, the stages of the evidence-identification process produce an indication of the level exhibited in that performance.

At the lowest level of the figure is a raw data stream, consisting of interface-specific and sometimes current-situation-specific locations, times, and durations of clicks, hovers, drag & drops, etc. These are vital to the simulation’s calculations, but it is not the terms in which students think when they are playing. They are not even aware of this level of activity taking place “under the hood” of the game. Nor do they need to be.

The next level up is so-called verb clauses in the game: Locations, times, durations, and objects of actions that are meaningful in the game semantics, such as “bulldoze this building” or “query pollution map.” These are the terms students *do* think in. This is the level of the data snippet in Fig. 3.

The next level higher is strategic patterns of verb clauses; e.g., build a new low-pollution plant before you bulldoze an old high-pollution one. Identifying such patterns in evolved situations where they are appropriate is data, clues, about a student’s level of thinking about the city’s underlying pollution & jobs system. Some of these were hypothesized, then later fine-tuned with pilot data, using the theory of the proficiencies and the design of the game. Others were developed subsequently through data mining (DiCerbo et al., 2007).

**Fig. 12:**
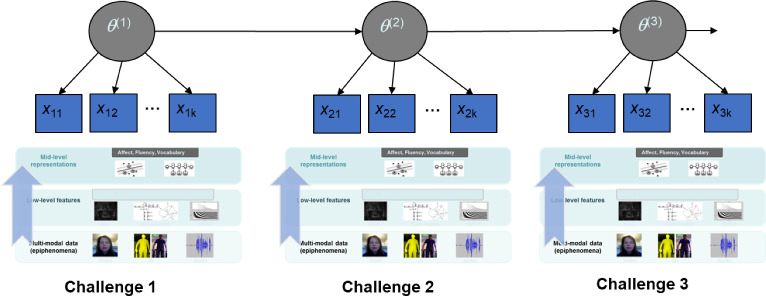
Latent variable measurement model for SimCityEDU.

Final challenge solutions and summary functions of strategic pattern usage in the challenge—counts, variety, and effectiveness as examples—were the vector of input variables *X* to the Bayes net psychometric model described next.

### Measurement Modeling

The preceding section described hierarchical evidence-identification processes within each *SimCityEDU* challenge *t*, culminating in a vector of variables $X_{t}$ that provide evidence about the level of thinking a student displayed in that performance. Figure 12 shows hierarchical evidence-identification chains for three successive challenges, for problems with increasingly complex underlying systems in the city.

In each problem, a student’s working through the task provides a vector of data summary variables $X_{t}$ at Challenge *t*, to reflect a student’s level of system thinking during that challenge. Recall that the underlying system and problem to solve in a challenge were designed to require thinking at targeted levels as described in the learning progression (Table 1). The values of these *X* variables were modeled in terms of conditional probabilities given $\theta$, as probabilities corresponding to expected performance by a student with proficiency at each given level in the learning progression. A student’s level in the progression variable $\theta$ was modeled as constant within a challenge but updated when the student moved on to the next challenge in accordance with probabilities at or above the level of the challenge crafted for a particular level in the learning progression.

As Fig. 10 suggests, there are a range of data analytic methods for identifying and evaluating evidence about a test-taker’s capabilities from *within* a complex performance (Yan et al., 2020). Some involve probability-based modeling, others do not. There are, however, advantages to using probability-based latent variable models to synthesize such evidence *across* tasks, modes of performance, or segments within larger performances when test-takers experience different pathways or subtasks.

First, evidence about capabilities is synthesized in terms of posterior distributions for the proficiency variables $\theta$. This approach subsumes traditional thinking about scores and measures but goes beyond it in ways that more complex performances and more ambitious inferences require (Mislevy and Gitomer, 1996; Rahimi et al., 2023). Further, validation approaches for examining the quality of “as if” reasoning about constructs that have developed over decades apply (Cronbach, 1971; Kane, 2006) and extend to new forms of data types and assessments (Ercikan et al., 2017; Zumbo et al., 2017)). Probability modeling also affords real-time updating, quantification of evidence through posterior distributions for $\theta$s, and, to investigate alternative explanations, model-critiquing methods with respect to individuals, groups, tasks, and background information.

## Concluding Statement

This article sketches a view of educational measurement that adapts concepts and tools that originated under trait, behavioral, and information-processing perspectives on assessment, but as reconceived and extended along lines prompted by the more encompassing sociocognitive perspective. The complementary structuring draws on tools and concepts from evidentiary argumentation. The pillars of the proposed approach are as follows:

- Whether explicit or implicit, the psychological/social underpinning and substance of an assessment are essential to interpreting and using measurement model elements.
- A sociocognitive complex adaptive systems perspective connects disciplines involved in learning and assessment. These include technology, analytics, learning science, domain-based research, automated scoring, and assessment design.
- Measurement modeling remains useful in designing, critiquing, and using educational assessments—for managing issues of evidence and inference—but its design and use acquire situated meaning in and through sociocultural milieus.
- Argumentation structuring provides a framework for integrating and for working through the practical issues of assessment design, critique, and use. This structuring incorporates the measurement modeling framework.

This necessarily brief article offers an initial view of a sociocognitive approach to educational measurement, and the pillars above are more in the character of assertions than conclusions from the preceding discussion. Fuller details appear in the *Sociocognitive Foundations of Educational Measurement* book mentioned in the introduction. But even that is more an explication than a dialog with alternative views on the nature of constructs and variables, the nature and role of probability models, the sociohistorical nature of what people learn in cultures, the nature and acquisition of their capabilities, the sociocognitive interplay between inter- and intra-individual phenomena in a society, and the relations among these. This work is needed.

As I write, however, I can say that this argument structuring and sociocognitive perspective offer insights into familiar assessment and measurement practices. They make explicit evidentiary reasoning principles that appear to underlie familiar practices that worked well in the environment in which they evolved. I and others are finding that these principles, understood beyond the particular forms they took, can be extended to incorporate advances in technology, analytics, and the psychology of learning.
